# Improving Prediction of Stress-Related Psychiatric Disorders through Computational Psychiatry

**DOI:** 10.1192/j.eurpsy.2026.11878

**Published:** 2026-07-31

**Authors:** I. Marinić, L. Mužinić Marinić

**Affiliations:** Department of psychiatry, University hospital Dubrava, Zagreb, Croatia

## Abstract

**Introduction:**

Stress-related disorders, including adjustment disorder, acute stress disorder, and post-traumatic stress disorder, arise from complex interactions of biological, psychological, and environmental factors. Computational psychiatry offers new opportunities by integrating heterogeneous data to build predictive models of risk and clinical outcomes.

**Objectives:**

This review explores the potential of computational methods to improve prediction of stress-related psychiatric disorders. The aim is to consider how these approaches may enhance early identification, prognosis, and support future clinical application.

**Methods:**

A narrative review was conducted using PubMed and Scopus, covering studies published between 2020 and 2025. Relevant studies applying computational methods to prediction and prognosis in stress-related disorders were analyzed.

**Results:**

Studies suggest that computational approaches hold promise for improving the prediction of stress-related psychiatric disorders. While findings remain preliminary, studies indicate the potential of these methods to capture complex interactions of risk factors.

**Conclusions:**

Computational psychiatry is a novel area of research with potential to advance early identification and prognosis in stress-related disorders. Further research is required to strengthen the validity of existing models and to support their eventual translation into clinical practice.

**Disclosure of Interest:**

None Declared

